# Factors analysis on the use of key quality indicators for narrowing the gap of quality of care of breast cancer

**DOI:** 10.1186/s12885-019-6334-5

**Published:** 2019-11-12

**Authors:** Chao Wang, Xi Li, Shaofei Su, Xinyu Wang, Jingkun Li, Xiaoqiang Bao, Meina Liu

**Affiliations:** Department of Biostatistics, School of Public Health, Harbin Medical University, No.157 Baojian Road, Harbin, 150081 China

**Keywords:** Breast cancer, Comprehensive evaluation, Single indicator evaluation, Influence factor

## Abstract

**Background:**

There are differences in the quality of care among breast cancer patients. Narrowing the quality differences could be achieved by increasing the utilization rate of indicators. Here we explored key indicators that can improve the quality of care and factors that may affect the use of these indicators.

**Methods:**

A total of 3669 breast cancer patients were included in our retrospective study. We calculated patient quality-of-care composite score based on patient average method. Patients were divided into high- and low-quality groups according to the mean score. We obtained the indicators with large difference in utilization between the two groups. Multilevel logistic regression model was used to analyze the factors influencing quality of care and use of indicators.

**Results:**

The mean composite score was 0.802, and the number of patients in the high- and low-quality groups were 1898 and 1771, respectively. Four indicators showed a difference in utilization between the two groups of over 40%. Histological grade, pathological stage, tumor size and insurance type were the factors affecting the quality of care. In single indicator evaluation, besides the above factors, age, patient income and number of comorbidities may also affect the use of these four indicators. Number of comorbidities may have opposite effects on the use of different indicators, as does pathological stage.

**Conclusions:**

Identifying key indicators for enhancing the quality-of-care of breast cancer patients and factors that affect the indicator adherence may provide guides for enhancing the utilization rate of these indicators in clinical practice.

## Background

Breast cancer is one of the most common malignant tumor in women worldwide [1], and remains a major public health issue in developed and developing countries [2, 3]. Treating breast cancer based on clinical practice guidelines can reduce the likelihood of cancer recurrence, increase survival, improve quality of life and reduce patient mortality [4, 5]. However, a wide gap still exists between the optimal recommended care for breast cancer and actual practice [6, 7]. For example, the rates of image-guided core needle biopsy and treatment with four cycles of adjuvant chemotherapy are only 34.1 and 12.1%, respectively, in China [8, 9]. A German study reported approximately 20% of patients treated with neoadjuvant therapy in the years 2009–2011 from a cohort of 39,570 patients and the treatment was recommended by the European Society of Breast Cancer Specialist (EUSOMA) [10, 11]. To ensure that actual treatment follows clinical guidelines, many institutions and professional organizations in various countries and regions have initiated great efforts to develop quality indicators for breast cancer and have applied these indicators to evaluate and monitor the quality of breast cancer care [12–15]. Quality indicators for breast cancer care can be used as a quality measurement tool for breast cancer care, and the use of these quality evaluation indicators can help identify deficiencies in the treatment process.

In the evaluation using these quality indicators, quality of care are reflected in the performance of indicators; the more indicators are completed, the better quality of care for patients [16]. In some studies, researchers have focused on the quality indicators for which the utilization rate is lowest in the patient population [17–19]. However, the indicators may be difficult to carry out in clinical practice. For example, the implementation of breast-conserving surgery is hampered by a shortage of trained radiation oncologists and technologists [8]. Instead, more attention should be paid to the indicators that the utilization rate varied largely between healthcare providers. The large rate gap indicates large room for improvement. These indicators are more likely to be key indicators with a large impact on quality of care.

The ability to successfully achieve all recommended indicators may be affected by many factors. For example, in breast-conserving therapy (BCT), which has shown increased survival rates compared with mastectomy, factors such as age, geographic location and payer status have been observed to influence the use of BCT [20]. Endocrine therapy, which may improve outcomes for breast cancer, may be affected by demographic, clinical and financial factors such as income and psychosocial factors like fear of toxicities [21, 22]. In addition, the physiological status of women at the time of breast cancer diagnosis can influence the choice for standard radiation treatment [23]. Identifying the factors that influence the application of these treatments is one of the ways to help improve the utilization rate of these indicators.

The objective of this study was to explore some indicators that can effective improve the quality of care and analyze the factors that may affect the use of these indicators. Narrowing the differences in treatment among patients may improve the overall quality of care of breast cancer patients.

## Methods

### Data sources

The process of data collection was similar to our previous study [24]. Information on patient demographics, tumor characteristics, diagnosis and treatment of breast cancer as well as data elements essential for identifying eligible patients for use of each treatment were extracted from medical records. Based on the quality of care indicators of breast cancer, a questionnaire was designed and data were collected by professionals using the questionnaire (see Additional file 1). Data were collected from medical records of patients diagnosed with invasive breast cancer in 10 tertiary hospitals, including three specialized tumor hospitals and seven general hospitals. A total of 4454 patients with primary invasive breast cancer (identified by the International classification of disease version 10 diagnosis codes: C50.902, C50.151, C50.251, C50.351, C50.451, C50.551) received all or part of their first course treatment in treating hospitals between June 2011 and June 2013. We excluded cases with breast cancer recurrence, bilateral breast cancer and distant metastasis of advanced breast cancer and patients missing information on tumor size and other pathological information to obtain a total of 3669 cases.

### Quality of care indicator

Twenty-three of the quality indicators that were previously developed by our research team were used in this study [25]. These indicators were examined throughout breast cancer care, from preoperative diagnosis to postoperative adjuvant therapy. The indicators are listed in Table 1. The denominator of the indicator denotes patients who were eligible without contraindications for the treatment, and the numerator denotes eligible patients who actually received the treatment.

**Table 1:** Definition of quality indicators for surgical care of breast cancer

Title	Quality indicators
Process	
1	Breast cancer patients who received mammography or breast ultrasound before surgery
2	Breast cancer patients who had diagnosis in cytology and/or histology before surgery
3	Breast cancer patients who received ER and PR assessment before systemic therapy
4	HER2 assessment before systemic therapy
5	Stage I-II breast cancer patients who underwent breast-conserving surgery
6	Breast cancer patients who received sentinel lymph nodes biopsy
7	Breast cancer patients who received axillary lymph nodes dissection
8	Premenopausal breast cancer patients who were administrated adjuvant chemotherapy
9	Postmenopausal breast cancer patients with high risk who received Adjuvant chemotherapy
10	Breast cancer patients who were administrated at least four cycles of Adjuvant chemotherapy
11	Breast cancer patients treated by trastuzumab in whom heart function was monitored every 3 months
12	Breast cancer patient whose radiotherapy treatment was completed within a 7-week interval from the initiation of radiotherapy after breast-conserving surgery
13	Breast cancer patients who received standard dose of radiotherapy at the whole breast after breast-conserving surgery
14	Breast cancer patients who received adjuvant radiotherapy at chest wall
15	Breast cancer patients who received tamoxifen or aromatase inhibitor treatment
16	Breast cancer patients who received neo-adjuvant chemotherapy
17	Breast cancer patients with hormone receptor status of the tumor stated in Pathology report
18	Breast cancer patients with pathology report stated category of primary tumor and regional lymph nodes with histologic grade
19	Breast cancer patients with pathology report stated number of Examined lymph nodes and positive nodes
20	Breast cancer patients with hormone receptor status of the tumor Stated in pathology report
21	Breast cancer patients with tumor size documented in pathology report
Management of symptoms or treatment toxicity	
22	Breast cancer patients who were administrated potent anti-emetic therapy
Communication and Cooperation	
23	Breast cancer patients who were recommended for five-year endocrine treatment

### Study variables

Baseline demographic information was obtained from the medical history records. Patient characteristics include age at diagnosis (< 40, 40–50, 50–60, > 60 years), types of insurance (Urban Resident Basic Medical Insurance (URBMI), Urban Employed Basic Medical Insurance (UEBMI) New Rural Cooperative Medical Scheme (NCMS)), income level, comorbidity (0, 1, ≥2), histological grade (high, moderately, poorly differentiated), cancer stage (I, II, III) and tumor size (< 2 cm, 2–5 cm, > 5 cm). Since information on patient income could not be gathered, as an alternative, area-level annual per capita income was extracted from the statistical bulletin of the regional economy and society developed in 2012; income level was classified into lower income (*<* 24,565 RMB) and higher income (≥24,565 RMB) groups, according to the national annual per capita income in 2012. Hospital characteristic refers to the type of hospital and included specialized tumor hospital and general hospital.

### Statistical methods

We used 23 indicators in the set of quality indicators for breast cancer care. To evaluate the patients’ comprehensive quality-of-care and its variation, we calculated the composite score of the patient’s treatment quality based on patient average method. The score was a simple ratio of the number of indicators for which care was provided divided by the number of indicators for which care should have been provided [26]. According to the mean score of patient composite score, we divided patients into the high- and low- quality groups. Baseline characteristics of the composite score were compared with ANOVA test. Baseline characteristics across different quality groups were compared with Chi-squared test or Kruskal-Wallis H test depending on the type of variable.

To obtain the indicator with the great degree of change of the utilization rate, we calculated the utilization rate of each quality indicator of the high- and the low- quality group and the difference of rate. The utilization rate was presented as a proportion of the sum of patients receiving care (numerator) to the total number of patients eligible for the care (denominator) [27].

Multilevel logistic regression model was used to analyze the factors that affect the use of the indicators that have great degree of change of the utilization rate. All statistical analyses were performed with SAS version 9.3 (SAS Institute, Cary, NC, USA). Statistical significance was set at *P* ≤ 0.05 and all statistical tests were two-sided.

## Results

### Treatment quality score of breast cancer patients

Our study included a total of 3669 breast cancer patients. The mean patient score of the patient’s treatment quality was 0.802. Patients were divided into the high-quality group (1898 cases) and the low-quality group (1771 cases) according to the mean score. The mean comprehensive scores of treatment quality in the two groups were 0.89 ± 0.06 and 0.70 ± 0.08, respectively. The baseline characteristics of patients according to the score of quality of care are listed in Table 2. The differences in the comprehensive score of types of insurance, income level, number of comorbidities, histological grade, stage and tumor size were statistically significant between the two patient groups. The characteristics of patients in the low- and high-quality groups are listed in Table 2. All variables except age at diagnosis were statistically significant.

**Table 2:** Comparisons of baseline characteristics between the low- and high-quality groups and the composite score among baseline categories^a^

Variable	Overall *N*(%)	Score of quality ($\overline{X}\pm S$)	*P* value	Quality-of-care group		*P* value
				Low quality (*N* = 1771) *n* (%)	High quality (*N* = 1898) *n* (%)	
Age			0.1584			0.2705
< 40	422 (12.05)	0.80 ± 0.12		212 (11.97)	230 (12.12)	
40–50	1414 (38.54)	0.80 ± 0.12		708 (39.98)	706 (37.20)	
50–60	1271 (34.64)	0.81 ± 0.12		588 (33.20)	683 (35.99)	
> 60	542 (14.77)	0.80 ± 0.12		263 (14.85)	279 (14.70)	
Comorbidities			< 0.0001			< 0.0001
0	2934 (79.97)	0.80 ± 0.12		1470 (83.00)	1464 (77.13)	
1	565 (15.40)	0.82 ± 0.11		235 (13.27)	330 (17.39)	
≥ 2	170 (4.63)	0.82 ± 0.13		66 (3.73)	104 (5.48)	
Types of insurance			< 0.0001			0.0026
NCMS	1216 (33.14)	0.79 ± 0.12		631 (35.63)	585 (30.82)	
URBMI	181 (4.93)	0.79 ± 0.13		94 (5.31)	87 (4.58)	
UEBMI	2272 (61.92)	0.81 ± 0.12		1046 (59.06)	1226 (64.59)	
Income			< 0.0001			< 0.0001
< ¥ 24,565	3135 (85.45)	0.78 ± 0.12		1688 (95.31)	1447 (76.24)	
≥ ¥ 24,565	534 (14.55)	0.89 ± 0.10		83 (4.69)	451 (23.76)	
Grade			< 0.0001			< 0.0001
High differentiated	185 (5.04)	0.81 ± 0.11		90 (5.08)	95 (5.01)	
Moderate differentiated	2481 (67.62)	0.81 ± 0.12		1104 (62.34)	1377 (72.55)	
Poor differentiated	558 (15.21)	0.80 ± 0.12		288 (16.26)	270 (14.23)	
Unknow	445 (12.13)	0.75 ± 0.12		289 (16.32)	156 (8.22)	
Stage			< 0.0001			< 0.0001
I	1229 (33.50)	0.77 ± 0.11		753 (42.52)	476 (25.08)	
II	1610 (43.88)	0.82 ± 0.12		638 (36.02)	972 (51.21)	
III	830 (22.62)	0.81 ± 0.12		380 (21.46)	450 (23.71)	
Tumor size			0.0047			0.0246
< 2 cm	2098 (57.18)	0.80 ± 0.12		1045 (59.01)	1053 (55.48)	
2-5 cm	1487 (40.53)	0.81 ± 0.12		690 (38.96)	797 (41.99)	
> 5 cm	84 (2.29)	0.83 ± 0.12		36 (2.03)	48 (2.53)	
Type of hospital			< 0.0001			< 0.0001
General hospital	1320 (35.98)	0.76 ± 0.11		861 (48.62)	459 (24.18)	
Specialized hospital	2349 (64.02)	0.82 ± 0.12		910 (51.38)	1439 (75.82)	

*Abbreviations*: *NCMS* New Rural Cooperative Medical Scheme, *URBMI* Urban Resident Basic Medical Insurance, *UEBMI* Urban Employed Basic Medical Insurance

^a^Discrete variables were expressed as counts (%) and continuous variables were expressed as a mean ± range

### Single indicator evaluation

**Fig. 1:**
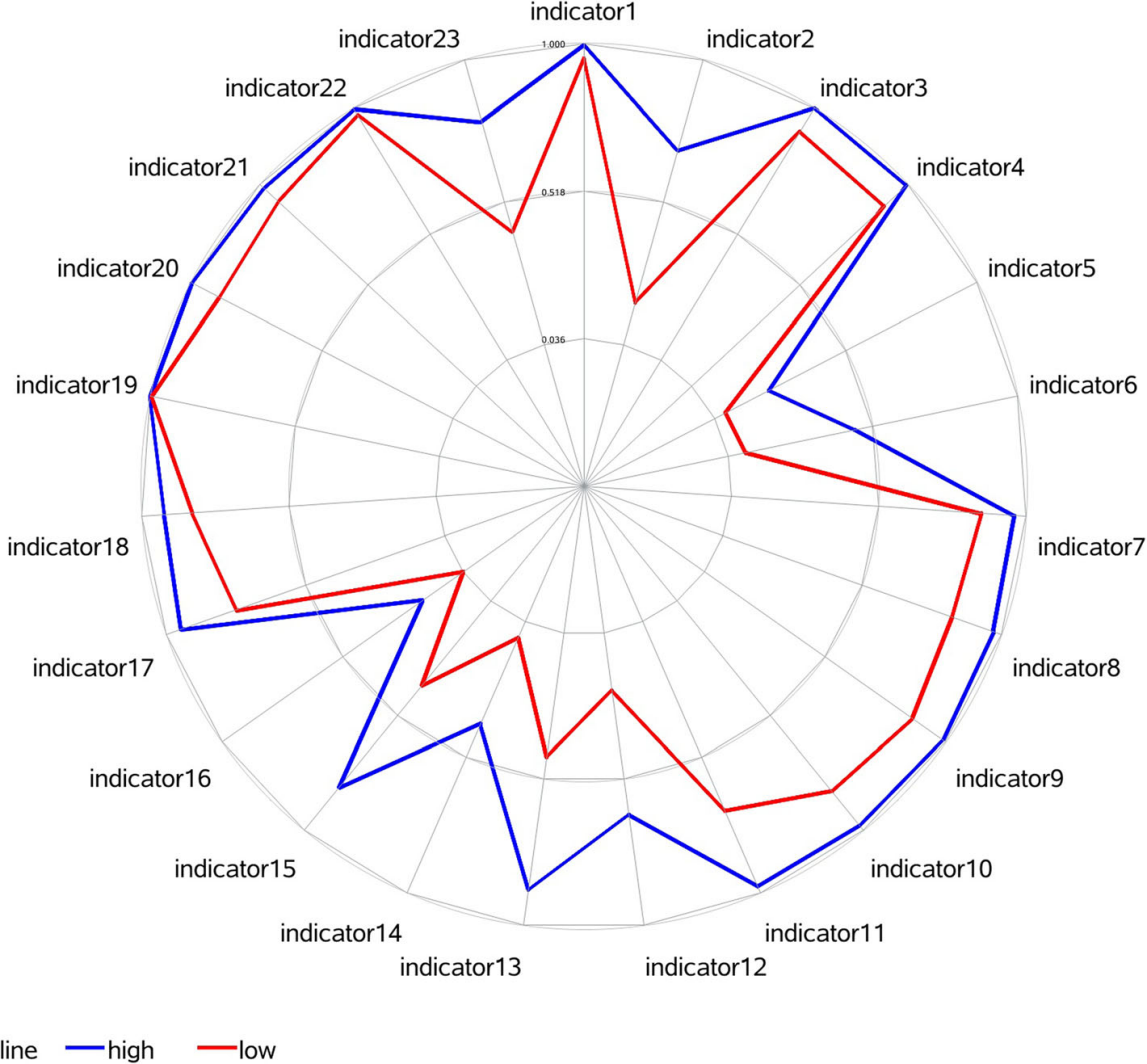
Utilization rate of the quality-of-care for breast cancer in two groups by radar chart. The blue line represents the high-quality group, the red line represents the low-quality group

We constructed a radar chart (Fig. 1) to show the utilization rate of the quality of care for breast cancer in the high- and low-quality patient groups. Four indicators showed a utilization rate with a difference of over 40% between the high quality and low-quality groups. Indicator 2 (breast cancer patients who had diagnosis in cytology and/or histology before surgery) showed the greatest difference (51.72%), followed by indicator 13 (proportion of breast cancer patients who received standard dose of radiotherapy at the whole breast after breast-conserving surgery) at 43.64%, indicator 15 (breast cancer patients who received tamoxifen or aromatase inhibitor treatment) at 42.84% and indicator 12 (treatment was completed within a 7-week interval from the initiation of radiotherapy after breast-conserving surgery) at 40.94%.

Multilevel logistic regression analysis was performed to determine the factors that affected the quality of care, and the results are shown in Table 3. Compared with patients with stage I, high differentiated and NCMS, those whose pathological stages were stage II, stage III, histological grades were moderately and poorly differentiated, and insurances were urban insurance may be more likely to have high quality of care. Compared with patients with a tumor size < 2 cm, patients with a tumor size of 2~5 cm may get low quality of care.

**Table 3:** Factors that affect the quality of care in Multilevel logistic regression model^a^

Variables	OR^a^ (95%CI)	*P* value
Age		
40–50	0.933 (0.715–1.216)	0.5939
50–60	1.152 (0.876–1.513)	0.2969
> 60	1.202 (0.864–1.672)	0.2617
< 40	Ref.	
Complications		
1	1.162 (0.913–1.480)	0.2058
≥ 2	1.272 (0.833–1.942)	0.2463
0	Ref.	
Types of insurance		
URBMI	0.924 (0.608–1.405)	0.6943
UEBMI	1.352 (1.125–1.625)	0.0032
NCMS	Ref.	
Income		
≥ ¥ 24,565	9.382 (0.973–90.467)	0.0521
< ¥ 24,565	Ref.	
Grade		
Moderate differentiated	1.661 (1.127–2.448)	0.0126
Poor differentiated	1.621 (1.039–2.528)	0.0347
Unkown	0.635 (0.409–0.985)	0.0432
high differentiated	Ref.	
Stage		
II	5.100 (3.995–6.512)	< 0.0001
III	3.866 (2.956–5.058)	< 0.0001
I	Ref.	
Tumor size		
2~5 cm	0.487 (0.392–0.607)	< 0.0001
> 5 cm	0.846 (0.476–1.501)	0.5447
< 2 cm	Ref.	
Type of hospital		
Specialized hospital	2.901 (0.396–21.233)	0.2383
General hospital	Ref.	

All variables in the Multilevel logistic regression analysis were adjusted for each other

^a^*Abbreviations*: *OR* odds ratio, *NCMS* New Rural Cooperative Medical Scheme, *URBMI* Urban Resident Basic Medical Insurance, *UEBMI* Urban Employed Basic Medical Insurance

Multilevel logistic regression analysis was used to analyze the factors influencing whether these indicators were used and the results are shown in Table 4. Preoperative biopsy (the use of indicator 2) was more likely for patients who had more comorbidities, lower histological grade, high insurance reimbursement, large tumor size and pathologic stage II or III (all *P* **<** 0.05). Patients who were younger or had pathologic stage I were more likely to receive treatment within a 7-week interval from the initiation of radiotherapy (all *P* **<** 0.05). Compared with patients with pathologic stage I, patients with stage II tumors may not receive standard dose of radiotherapy at the whole breast after breast-conserving surgery (all *P* **<** 0.05). Complete endocrine therapy was more likely for patients who had less comorbidities, pathologic stage III and higher insurance reimbursement and income (all *P* **<** 0.05).

**Table 4:** Factors that affect the use of four indicators in Multilevel logistic regression model^a^

Variables	Indicator 2	Indicator12	Indicator13	indicator 15
	OR(95%CI)	OR(95%CI)	OR(95%CI)	OR(95%CI)
Age				
40–50	0.807 (0.608–1.071)	0.468 (0.125–1.753)	0.862 (0.325–2.282)	0.943 (0.693–1.282)
50–60	0.850 (0.635–1.138)	0.203 (0.044–0.928)	0.676 (0.228–2.001)	1.219 (0.882–1.684)
> 60	0.718 (0.501–1.029)	2.339 (0.168–32.547)	0.865 (0.134–5.588)	1.299 (0.879–1.920)
< 40	Ref.	Ref.	Ref.	Ref.
Complications				
1	1.339 (1.036–1.731)	1.102 (0.175–6.922)	1.073 (0.220–5.231)	1.319 (0.982–1.773)
≥ 2	1.830 (1.166–2.874)	0.371 (0.014–9.767)	0.825 (0.057–12.013)	0.583 (0.358–0.950)
0	Ref.	Ref.	Ref.	Ref.
Types of insurance				
URBMI	1.386 (0.857–2.243)	1.409 (0.081–24.526)	4.376 (0.618–30.965)	1.354 (0.828–2.214)
UEBMI	1.357 (1.116–1.650)	1.173 (0.318–4.325)	2.232 (0.771–6.464)	1.873 (1.508–2.327)
NCMS	Ref.	Ref.	Ref.	Ref.
Income				
≥ ¥ 24,565	14.142 (0.384–520.935)	2.625 (0.014–506.325)	1.841 (0.064–52.931)	10.341 (1.161–92.116)
< ¥ 24,565	Ref.	Ref.	Ref.	Ref.
Grade				
Moderate differentiated	2.079 (1.394–3.100)	2.466 (0.430–14.139)	1.344 (0.310–5.830)	1.072 (0.709–1.619)
Poor differentiated	1.654 (1.035–2.643)	1.645 (0.223–12.109)	0.473 (0.080–2.798)	0.917 (0.544–1.547)
Unkown	3.277 (2.079–5.167)	1.462 (0.303–7.058)	0.876 (0.168–4.578)	1.406 (0.854–2.316)
High differentiated	Ref.	Ref.	Ref.	Ref.
Stage				
II	1.341 (1.046–1.718)	0.254 (0.079–0.813)	0.250 (0.091–0.685)	1.071 (0.818–1.402)
III	1.501 (1.136–1.984)	0.286 (0.041–1.996)	0.213 (0.035–1.308)	1.426 (1.053–1.932)
I	Ref.	Ref.	Ref.	Ref.
Tumor size				
2~5 cm/≥ 2 cm	1.626 (1.293–2.044)	0.667 (0.141–3.150)	1.146 (0.335–3.925)	1.011 (0.786–1.301)
> 5 cm	2.805 (1.403–5.606)	–	–	0.606 (0.270–1.360)
< 2 cm	Ref.	Ref.	Ref.	Ref.
Type of hospital				
Specialized hospital	4.726 (0.195–114.334)	1.035 (0.010–112.506)	11.045 (0.584–208.892)	2.648 (0.394–17.822)
General hospital	Ref.	Ref.	Ref.	Ref.

Missing data were characterized as unknown

For indicator 12, indicator 13, tumor sizes was divided into < 2 cm and ≥ 2 cm; for indicator 2 and indicator 15, tumor sizes was divided into < 2 cm,2~5 cm and ≥ 5 cm

All variables in the Multilevel logistic regression analysis were adjusted for each other

^a^*Abbreviations*: *OR* odds ratio, *NCMS* New Rural Cooperative Medical Scheme, *URBMI* Urban Resident Basic Medical Insurance, *UEBMI* Urban Employed Basic Medical Insurance

## Discussion

The quality of high- and low- quality group was obtained through comprehensive evaluation; we identified the four indicators that showed a large difference in their application between the patient groups and we analyzed the factors influencing the use of these indicators. In this study, we focused on the indicators that showed large variations in applications between high and low groups rather than indicators that were less frequently applied in both groups. The indicators which utilization rate are both low in two groups means the variations of utilization rates are little, indicated that in clinical practice they are difficult to complete among patients. In our study, the two indicators with the worst completion, in which the utilization rates were less than 20%, included early stage breast cancer patients who underwent breast-conserving surgery and received neo-adjuvant chemotherapy and variation of these indicators utilization rate are both less than 15%. Neoadjuvant chemotherapy and breast-conserving surgery have been a trend in breast cancer care, but these treatments require an established integrated multidisciplinary care strategy that includes pathologists, radiologists, surgeons and oncologists. Implementation of these treatments is also hampered by a shortage of trained medical staff [8], and thus even if we want to improve the utilization rate of indicators, it may be difficult to substantial increase their use. We calculated the utilization rate of the two indicators for each year from 2011 to 2013. The utilization rates of both indicators were very low in the 3 years and there was no obvious trend over time. The rates of breast-conserving surgery from 2011 to 2013 were 13.71, 13.42, 13.58% respectively. And the rates of neo-adjuvant chemotherapy from 2011 to 2013 were 11.72, 13.92, 13.68% respectively. We may conduct a further study on how to improve the indicators with low utilization rate in the future.

The four indicators selected in the current study, in which the variations of utilization rate were more than 40%, may be key indicators that lead to differences in quality of care. Because the variations of utilization rate are so great, there is tremendous room for improvement in the use of these indicators and the application could be improved relatively easily. Identification of the factors that may affect the use of these indicators may provide some suggestions for clinical improvement of quality of care. If doctors strictly followed clinical guidelines, the utilization of preoperative biopsy may be improved [28, 29], which was consistent with our results. In another study, cancer survivors who reported financial problems were also more likely to report delayed medical care or foregoing medical care and prescriptions [30–32], which was consistent with our results. For patients who received radiotherapy after breast-conserving surgery, factors such as pathological stage, and age at diagnosis may affect the application of complete radiotherapy in a standard dose and treatment within a 7-week interval.

In our study, the same factor may have opposite impacts on the use of different indicators. For patients with a relatively late pathological stage, it is more possible to conduct preoperative biopsy to understand the disease development [33], and to conduct endocrine therapy to inhibit the growth of cancer cells [30, 32]. However, our study showed that these patients are less likely to have postoperative radiotherapy after breast-conserving surgery. Similarly, for patients with more comorbidities of basic diseases, doctors are more inclined to perform preoperative biopsy to choose a better treatment plan for subsequent treatment [29]. However, the side effects of endocrine treatment will aggravate patient conditions with other more basic diseases, and the treatment is difficult for most patients to endure, which will make it difficult for patients to complete endocrine treatment [34–36]. Therefore, in cases in which there are contradictions with use of indicators, it is important for doctors to weigh the advantages and disadvantages and try to select and achieve the indicators that are most suitable for patients.

This study had several limitations. First, when analyzing the single indicator influencing factors, there were no pathological stage I patients take mastectomy, we take stage II as a reference; because of the sample is a little small, for radiotherapy after BCT, fewer patients’ tumor are greater than 5 cm and therefore we divided tumor sizes into < 2 cm and ≥ 2 cm. Second, we conducted observational research and therefore we cannot prove causation between the use of indicators and characteristics of patients. Third, we didn’t collect information of the reasons why patient didn’t receive certain therapy or diagnostics from the medical reports, which may influence the quality scores. Finally, the breast cancer patients included in this study had invasive breast cancer. The factors that may affect quality of patients and the use of indicators may not be applicable to other types of breast cancer and other cancers.

## Conclusions

Here we identified key indicators which have variation of indicators utilization rate between different quality of patients could enhance the quality of care breast cancer patients. Analysis of the factors that affect the indicator adherence may provide some guides and suggestions for enhancing the utilization rate of these indicators in clinical practice.

## Supplementary information

**Additional file 1.** Medical record questionnaire for breast cancer patients.

## Data Availability

The data that support the findings of this study are available from ten teaching grade A tertiary hospitals located in north of China but restrictions apply to the availability of these data, which were used under license for the current study, and so are not publicly available. Data are however available from the corresponding authors upon reasonable request and with permission of those investigated hospitals.

## Abbreviations

BCT: Breast-conserving therapy
EUSOMA: European Society of Breast Cancer Specialist
NCMS: New Rural Cooperative Medical Scheme
UEBMI: Urban Employed Basic Medical Insurance
URBMI: Urban Resident Basic Medical Insurance
