# Supplementary material for: Factors analysis on the use of key quality indicators for narrowing the gap of quality of care of breast cancer
Source: BMC Cancer. 2019 Nov 12;19:1099. doi: 10.1186/s12885-019-6334-5 (PMC6852954; doi:10.1186/s12885-019-6334-5)
Supplement: Supplementary file 1 — Additional file 1. Medical record questionnaire for breast cancer patients. [file 12885_2019_6334_MOESM1_ESM.docx]

**Additional file 1**

**Medical record questionnaire for breast cancer patients***

**Demographic information and Tumor characteristics**

1. Patient ID: ;

2. Age: ;

3. Marital status: ①single; ②married; ③divorced; ④widowed;

4. Occupation: ①Clerks; ②Workers, farmers;③self-employed laborer, service; ④Others:____________;

5. Annual household income: RMB;

6. Residence: ①urban; ②rural;

7. Primary payer status: ①Urban Resident Basic Medical Insurance (URBMI); ②Urban Employed Basic Medical Insurance (UEBMI); ③New Rural Cooperative Medical Scheme (NCMS); ④Others:____________;

8. Admission date: ; length of hospital stay: days;

9. Comorbidities: ①none; ②have:

| ①hypertension | ②diabetes mellitus | ③tuberculosis | ④bronchitis |
| --- | --- | --- | --- |
| ⑤pneumonia | ⑥tuberculous pleurisy | ⑦coronary heart disease | ⑧heart failure |
| ⑨myocardial infarction | ⑩ angina | ⑪ stroke | ⑫ liver disease |
| ⑬historical cancer | ⑭others： | | |

10. Smoke: ① no; ② yes;

11. History of menstrual: menophania : years; menopause ① no; ② yes;

age of menopause ;

12. Family history of breast cancer : ①none; ②have;

13. Postoperative pathological report: ①none; ②have;

14. Primary lesion site: ①left; ②right; ③underarm; ④other;

15. Tumor size: __________cm;

16. Histological grade: ①High differential; ②Moderately differential; ③Low differential;

17. Histological classification: ①ductal carcinoma; ②Lobular carcinoma; ③medullary carcinoma; ④ other;

18.Distant metastases：①no; ②yes;

19.Tumor stage:

①clinical stage: T______; N_____; M_____;

②pathological stage: T______; N_____; M_____;

20. Axillary lymph nodes examination: ①negative; ②positive;

21. HER2 test：①negative; ②positive; ③did not test;

22. Estrogen receptor(ER) test：①negative; ②positive; ③did not test;

23. Progestrone receptor(PR) test：①negative; ②positive; ③did not test;

**Inspection item**

| Item name | inspection |
| --- | --- |
| 1. mammography before surgery | ①no; ②yes; data： ； |
| 2. breast ultrasound before surgery | ①no; ②yes; data： ； |
| 3. cardiac ultrasound before surgery | ①no; ②yes; data： ； |
| 4. cytology and/or histology before surgery | ①no; ②yes; data： ； |
| 5. Preoperative hollow needle biopsy | ①no; ②yes; data： ； |

**Therapy**

1.Surgery: ①no; ②yes;

Date of surgery: (yyyy/mm/dd);

Surgeon:

2.Preoperative communication: ①no; ②yes; ③not recorded;

3.Surgical procedures: ①breast-conserving surgery; ②mastectomy;

4.Sentinel lymph node biopsy：①no; ②yes;

Positive number/removed number of lymph nodes /

5.Axillary lymph node dissection：①no; ②yes;

Positive number/removed number of lymph nodes /

6.Resection margin status: ①negative; ②positive; ③did not recode;

7.Recommended for postoperative endocrine treatment: ①no; ②yes;

8.Postoperative adjuvant chemotherapy: ①no; ②yes;

Chemotherapist: ;

Department of chemotherapy: ①internal medicine department; ②radiology department; ③surgery department; ④other ;

Periodic number: ;

Scheme: ;

Data: ;

9.Neoadjuvant chemotherapy: ①no; ②yes;

Periodic number: ;

10.Postoperative radiation therapy: ①no; ②yes;

11. Potent anti-emetic therapy: ①no; ②yes;

Postoperative complication: ①no; ②yes:

| ①postoperative infection | ②hemorrhage | ③ radiation pneumonia |
| --- | --- | --- |
| ④emesis after chemotherapy | ⑤ myelosuppression | ⑥ others： |

**Outcome**

| 1. postoperative recurrence | ①no; ②yes; data： ； |
| --- | --- |
| 2. postoperative metastasis | ①no; ②yes; data： ； |

*To guarantee the validity and reliability of the questionnaire, we conducted a pilot test. During the data collection process, regular correspondence was maintained with those compiling the data to identify any ambiguities or deficiencies in the information collection to facilitate timely modification and accelerate the process of data extraction. Following the data collection, 5% of the records were randomly selected for a secondary data collection using methods identical to the first data collection, and the test-retest reliability was high (up to 95%).
